# Porous titanium granules in the treatment of peri-implant osseous defects—a 7-year follow-up study

**DOI:** 10.1186/s40729-017-0106-2

**Published:** 2017-12-04

**Authors:** Heidi Andersen, Anne Merete Aass, Johan Caspar Wohlfahrt

**Affiliations:** Department of Periodontology, Institute of Clinical Dentistry, University of Oslo, Pb. 1109 Blindern, 0317 Oslo, Norway

## Abstract

**Background:**

A great number of different treatment protocols for peri-implantitis have been suggested but there is no consensus regarding the most effective intervention. The aim of the present study was to evaluate the long-term clinical and radiographic results from a study on peri-implant osseous defect reconstruction.

Patients having participated in a randomized clinical study 7 years earlier were invited for a re-examination. The treatment procedures included open flap debridement (OFD) with or without defect reconstruction with porous titanium granules (PTGs). Clinical parameters (probing pocket depth and bleeding on probing) and radiographic measurements were registered.

**Findings:**

Of the original 32 patients, 12 patients with 12 implants were finally examined after 7 years (7.3 years [6.7–8]). Patients had been maintained one to two times yearly. The PTG group showed a mean probing pocket depth of 4.3 mm ± 2.4 compared with 3.5 mm ± 1.2 in the OFD group, at the deepest site. The change between the 12 months and the 7-year examination was similar in both groups.

Five of the test implants and five of the control implants had at least one site with positive bleeding on probing score.

The mean radiographic defect depth change as compared to 12 months was an increase of 1.9 mm ± 2.0 in the PTG group and a mean radiographic defect depth increase of 1.3 mm ± 1.4 in the OFD group.

Due to the small number of patients, a statistical analysis was not performed, but the results indicated a minimal difference in osseous defect depth as compared with baseline and between groups.

No PTG exposed to the oral cavity was observed, but the graft particles were seemingly scattered in the peri-implant soft tissue.

**Conclusions:**

This long-term follow-up of surgical treatment of peri-implant osseous defects showed unpredictable results.

## Findings

### Introduction

Peri-implantitis was suggested [1] as an infectious and pathological site-specific disease in surrounding peri-implant tissues.

At the 6th European Workshops on Periodontology, consensus was made on the definitions related to the peri-implant diseases, peri-implant mucositis and peri-implantitis, respectively [2].

Peri-implant mucositis describes an established inflammatory lesion in the soft tissue while peri-implantitis also affects the supporting bone [3].

The prevalence of peri-implantitis affects 20% of patients [4]. A vast number of different treatment protocols for peri-implantitis have been suggested through the years [5, 6].

Today there is no consensus regarding the most effective intervention. Current general understanding among both clinicians working in the field and within the research community is that surgical exposure of the implants and removal of the granulation tissue seem to be necessary [2]. A surgical strategy with or without bone recontouring to accomplish pocket elimination to optimize infection control seems to work in many cases [7].

In some selected cases with peri-implant bone loss, it may be considered to reconstruct the lost osseous lesion. The current scientific evidence available in the literature for the efficacy of reconstructive and surgical strategies in treating peri-implantitis is, however, limited [8].

Porous titanium granules (PTGs, Natix®, Tigran Technologies AB, Malmö, Sweden) were initially used in orthopaedics for stabilization of hip prostheses to enhance bone regeneration [9, 10].

Regarding oral and maxillofacial surgery, PTG was introduced as a bone graft substitute for use in sinus lifts [11].

A porous titanium granule is 700–1000 μm in diameter. The total titanium surface of the ultra-porous granules is approximately 2 cm^2^ [12], which provides a significant blood-to-titanium contact area. Titanium has also been demonstrated to be a potent activator of the blood coagulation system with thrombus formation, which may be interesting from the perspective of bone healing and osseous growth [13].

In 2011, Wohlfahrt et al. presented human histological support that re-osseointegration of a contaminated dental implant with peri-implantitis was biologically possible. Grafting of a peri-implant defect with PTGs may lead to newly formed bone both in close connection with the graft material as well as with the contaminated implant surface [14].

In 2012, the same group of researchers presented results from a randomized parallel arm case-control clinical study, using porous titanium granules as a bone substitute in the corrective surgical treatment of peri-implant osseous defects. Grafting of the defects with PTG was compared with open flap debridement alone. No clinical differences between groups were found after 12 months, but a better defect fill was seen on radiographs in the PTG group [12]. A multicentre study [15] reported similar results.

The aim of the present study was to evaluate the long-term clinical and radiographic results from a study on peri-implant osseous defect reconstruction.

### Materials and methods

Data from patients having participated in a randomized case-control clinical trial was screened with the aim to re-examine all treated implants. The present study was approved by the Regional Committee for Medical and Health Research Ethics, South East Norway (REK 2015/90).

Wohlfahrt and co-workers [12] described the study population and the 12-month results. In brief, 32 subjects diagnosed with peri-implantitis, fulfilling the inclusion criteria of having an implant demonstrating an intrabony component ≥ 4 mm, probing pocket depth (PPD) of at least 5 mm at one site with bleeding on probing (BoP) and/or suppuration were included, randomized to either the case or control group, treated accordingly and followed for 12 months. The prosthetic supraconstruction was removed and evaluated with respect to occlusal adjustments; in some cases, renewal was performed. Access for oral hygiene was evaluated and corrected if necessary. After the oral hygiene phase and any necessary active periodontal treatment, the study participants received surgical therapy. Sixteen individuals were randomly assigned to the case group and received reconstructive treatment of one implant each with PTG additional to surgical open flap mechanical and chemical debridement with titanium curettes and EDTA gel (PrefGel, Straumann Inc., Basel, Switzerland). The other 16 individuals were treated with open flap debridement alone. Implants were submerged for 6 months; thereafter, the prosthetic supraconstructions were repositioned. Results from the evaluation at 12 months were presented in a publication by Wohlfahrt et al. in 2012. Thereafter, the patients were discharged from the university clinic and maintenance, and follow-ups were handled by the referring dentists.

After 7 years (7.3 years [6.7–8]), all patients who completed the original study were invited for a re-examination. After a multitude of recall strategies including telephone and letters, 17 subjects responded and were recalled to the university dental clinic between April and November 2015. After signing the written informed consent, medical and dental history were recorded and a clinical examination and full-mouth radiographs were taken.

**Fig. 1:**
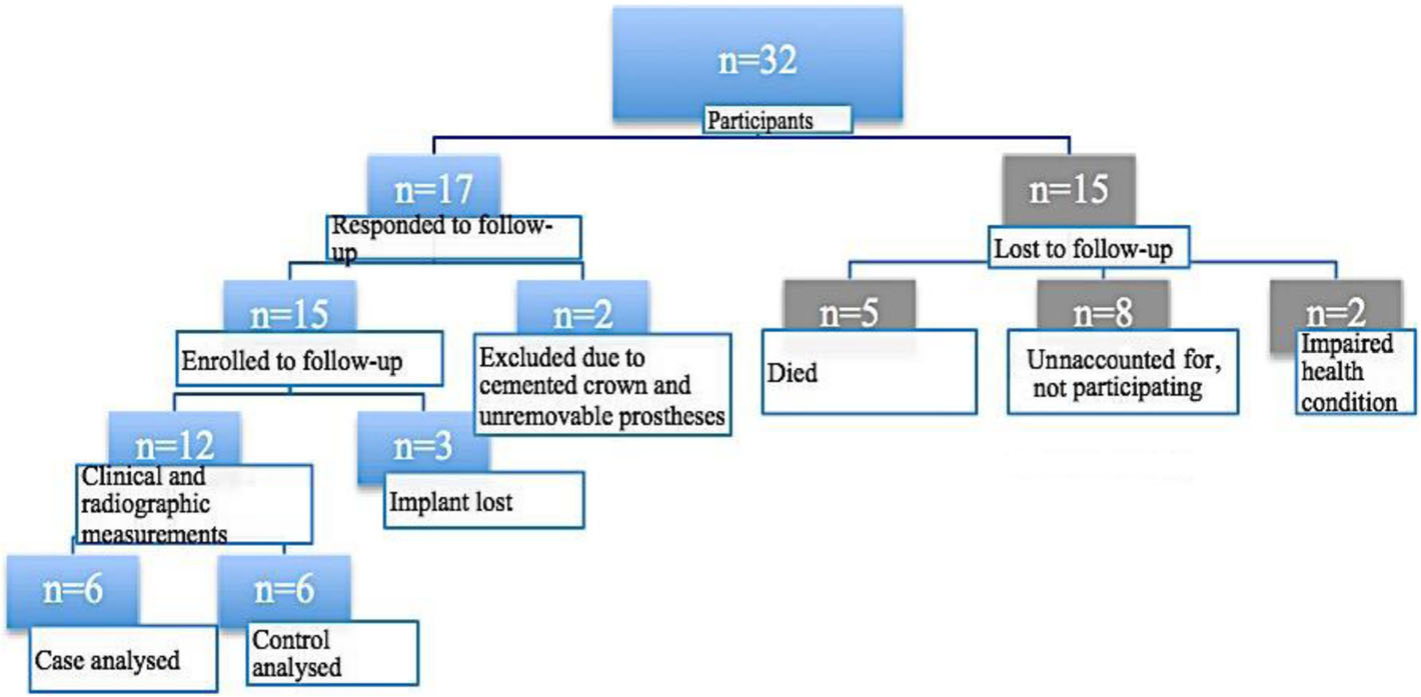
Consort flowchart

In total, 12 subjects completed baseline, 12-month and 7-year examinations and were thus included in this analysis (Fig. 1).

#### Clinical examinations

All clinical examinations were performed by the same calibrated, blinded and board-certified periodontist (AMA) who also had done the examinations in the previous study. The same clinical indexes and the same type of instruments as in the original study were used.

Dichotomous plaque scores and PPD were recorded at six sites per treated implant, using a 0.20-N (20-g) defined force periodontal probe (UNC, DB 764 R, AESCULAP, B. Braun). Dichotomous bleeding on probing (BoP) and dichotomous purulent suppuration (PuS) were all registered at six sites (i.e. mesiofacial, facial, distofacial, distolingual, lingual and mesiolingual). The height of the buccal keratinized mucosa was also registered.

One postgraduate student from the prosthodontic department evaluated the supraconstructions related to access for interproximal oral hygiene, misfit of prostheses, prosthetic material, number of implants supporting the fixed dental prostheses, presence of cantilever extensions and presence of abutments.

The occlusion, articulation, interferences, horizontal overbite, vertical overbite and occlusal morphology were examined and recorded.

Evaluation of the supraconstructions considering mechanical complications such as fractures of or material or chipping, abutment screw loosenings, abutment fractures, and implant and framework fractures were performed.

The prosthetic supraconstructions were carefully cleaned and positioned in ultrasound before repositioned on the implants.

#### Radiographic evaluation

Intraoral radiographs were obtained of the included implants and for the full dentition using Digora phosphor plates with a standard Eggen film holder (Eggen X-ray AS). The radiographs were scanned and digitized with the Digora Optime Soredex intraoral digital imaging system. The technique used at baseline and at the 12-month recall appointment, with an occlusal bite index with dental wax (Kerr Dental®) stuck to an Eggen film holder, had dried out and was no longer possible to use at the 7-year re-examination.

For each radiograph, the width of the implant was used for image calibration.

The defect height was measured from a well-defined reference point at the most coronal part of the implant body, on radiographs taken at baseline, 12 months and 7 years. The radiographic measurements were performed by one investigator (AMA), blinded to the examination timepoint. The changes in vertical defect depth were also calculated. To analyse the radiographs taken, Image J, 1.42q, US National Institutes of Health, Research Services Branch was used.

The radiographic measurements were repeated twice, after 1 week and after 10 weeks, and calculated with Pearson correlation coefficient. A strong positive correlation was demonstrated (*r* = 0.99–0.93).

#### Statistical analysis

The study population of the follow-up study did not reach statistical power due to the many drop-outs; the calculations were therefore limited to descriptive findings applied on the clinical and radiographic data assessed at baseline, 12-month examination and after 7 years.

Continuous variables were expressed as means and standard deviation.

### Results

Seven years (7.3 years [6.7–8]) after surgical treatment of peri-implantitis, 17 individuals met for the follow-up examination.

During 2008–2015, five subjects had died and ten subjects were lost to follow-up for various reasons. Among individuals accepting the invitation for the follow-up, three subjects, all in the PTG group, had lost their treated implants. Two subjects were excluded due to technical complications with the supraconstructions; one patient had received a new cemented single crown, and one subject had an overdenture impossible to remove.

In total, 12 individuals were finally included (Fig. 1).

The majority (10/12) of the subjects in the present study were smokers or former smokers, and 8/12 subjects had a history of periodontitis. The characteristics and demographic data of the groups are presented in Table 1. Most implants had screw-retained supraconstructions and had a mean function time of 14 years and were in the anterior region of the maxilla and mandibula.

**Table 1:** Characteristics of the subjects at the 7-year follow-up

Characteristic	Case (PTG) group (*n* = 6)	Control group (*n* = 6)
Age (year), mean ± SD	67 ± 12.9 range, 45–79	67.2 ± 11.8 range, 53–85
Male/female (*n*)	3/3	2/4
Smoker	2/6	3/6
Former smokers	3/6	2/6
Diabetes (type 2)	1/6	1/6
PlI at follow-up (%), mean ± SD	19.6 ± 15.5	28.8 ± 35.1
Periodontal supportive care	6/6	5/6
Reason for placing implants		
Periodontitis	5/6	3/6
Trauma	1/6	1/6
Caries	0	1/6
Agenesia/Anodontia	0	1/6

Three different manufactures represented the implants included in the analysis. Seven subjects had Brånemark implants, four subjects had been treated with Astra Tech implants and one subject had a Straumann implant. Eleven subjects had periodontal supportive care performed by their hygienist or general dentist at a frequency of 6 months to once yearly. One subject reported sporadic visits. About 50% of the subjects performed dental home care twice a day including tooth brushing and interdental cleaning. Access for oral hygiene procedures was deemed acceptable for all the supraconstructions in both groups. None of the re-assessed patients reported prosthodontic retreatment after completing the previous study. Technical complications were observed in two supraconstructions, one with fractured occlusal screw (bridge screw) and one presented a lost filling of the access hole.

#### Clinical and radiographic outcomes

Three implants in the PTG group were lost during the study period.

Two implants had to be excluded due to technical complications, one from each group.

The results thus refer to 12 implants, evenly distributed between the two treatment modalities.

The PTG group showed a mean PPD of 4.3 mm (± 2.4) at the deepest site compared with 3.5 mm (± 1.2) in the OFD group. The baseline mean PPD was 6.5 mm (± 1.9) in the PTG group and 6.5 mm (± 2.3) in the control group and at 12 months 4.9 mm (± 1.8) in the PTG group and 4.4 mm (± 4.4) in the control group.

Five of the test implants and five of the control implants had at least one site with positive BoP score. Suppuration was demonstrated at five implants, two in the PTG group and three in the OFD group. The plaque scores were slightly higher in the OFD group at the final examination (Table 2).

**Table 2:** Data at baseline, 12 months and 7 years for the PTG and OFD groups

Patient/implants	Radiographic defect height at deepest site (mm)			The site with the deepest PPD (mm)			PlI	BoP	BoP	BoP
	Baseline	12 months	7 years	Baseline	12 months	7 years	7 years	Baseline	12 months	7 years
1. PTG	5.0	2.3	1.6	6	3	2	0/6	6/6	6/6	0/6
2. PTG	5.6	3.9	5.6	10	7	4	1/6	6/6	6/6	6/6
3. PTG	7.0	3.5	7.0	9	7	9	2/6	6/6	6/6	6/6
4. PTG	3.1	1.7	2.5	6	4	4	0/6	6/6	0/6	6/6
5. PTG	4.6	0.8	2.2	9	7	3	0/6	4/6	6/6	6/6
6. PTG	7.1	4.1	8.9	8	4	4	6/6	3/6	4/6	3/6
Mean ± SD (min, max)	5.4 ± 1.5 (3.1–7.1)	2.7 ± 1.3 (0.8–4.1)	4.6 ± 3.0 (1.6–8.9)	8 ± 1.7 (6–10)	5.3 ± 1.9 (3–7)	4.3 ± 2.4 (2–9)	25%	92%	77%	75%
1. OFD	3.3	1.2	3.2	8	5	4	1/6	6/6	0/6	6/6
2. OFD	3.2	2.6	4.4	5	3	5	0/6	6/6	6/6	6/6
3. OFD	6.3	5.3	5.8	7	8	4	6/6	6/6	6/6	6/6
4. OFD	3.9	3.5	6.8	6	4	4	0/6	6/6	6/6	4/6
5. OFD	5.3	4.2	4.9	13	5	2	0/6	6/6	6/6	0/6
6. OFD	3.5	2.4	1.8	9	2	2	6/6	6/6	6/6	6/6
Mean ± SD (min, max)	4.3 ± 1.3 (3.2–6.3)	3.2 ± 1.4 (1.2–5.3)	4.5 ± 1.8 (1.8–6.8)	8 ± 2.8 (5–13)	4.5 ± 2.1 (2–8)	3.5 ± 1.2 (2–5)	36%	100%	83%	78%

The mean width of keratinized tissue for the PTG group and OFD group was 1.5 mm (± 1.0) and 2.8 mm (± 1.2), respectively. From baseline to the follow-up examinations presented here, the width of keratinized tissue decreased more in the PTG group as compared with the OFD group.

Radiographic findings demonstrated similar values after 7 years as the baseline regarding defect depth height in 6/12 treated implants. Three implants, one in the PTG group and two in the OFD group, had progression of radiographic defect depth height, measured at the deepest site, compared to baseline.

Assessing the results after 12 months, five of the implants in the PTG group and five of the implants in the OFD group had progression of radiographic defect depth height.

There was an aggravation in mean radiographic osseous defect level of 1.9 mm ± 2 and 1.3 mm ± 1.4 in the PTG group and OFD group, respectively.

Three implants demonstrated improved radiographic defect depth height, measured at the deepest site, at 7 years compared to baseline. Two implants represented the PTG group and one implant the OFD group.

**Fig. 2:**
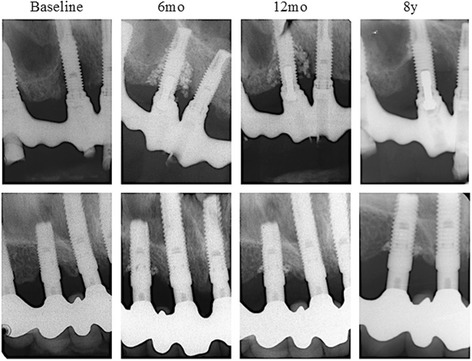
PTG observed in radiographs at the 8-year follow-up

The PTG graft particles were seemingly scattered, as judged by radiographs (Fig. 2). Loss of grafting material was extensive in two cases, and most grafting material was still present in the osseous defect in two cases. At test sites where the implant had been lost, some remaining PTG particles were seen in the surrounding tissue.

### Discussion

The aim of the study was to evaluate the long-term clinical and radiographic results from a study on peri-implant osseous defect reconstruction [12].

Minimal differences were observed when comparing the two groups at baseline, 12-month and the 7-year examinations, but the power to detect a true difference at the 7-year examination was small due to the low number of patients finally re-examined.

Most of the implants had a deeper radiographic defect depth after 7 years compared to the 12-month examination, and minimal differences were seen when comparing with baseline measurements. It is therefore important to consider the original osseous defect depth around treated implants when aiming to diagnose stable peri-implant conditions [16]. Moreover, three of the implants in the test group had lost all bony attachment and had been explanted. However based on the evaluation presented here, it was not possible to clarify if differences in patient-related factors such as the original defect depth or treatment modality were causative for this outcome.

Few studies report long-term results after treatment of peri-implantitis. In this context, it is important to remember that the only true end point for such therapies will be the loss of implants, but most studies solely report clinical and subclinical parameters which are only surrogate markers for the disease state and true result of the performed therapy [17].

In a meta-analysis by Khoskham et al., they report a mean radiographic defect fill of 2.41 mm after regenerative treatment of peri-implant osseous defects and a minimum healing time of 36 months [18].

Recently, Schwarz et al. reported on a 7-year follow-up on a peri-implantitis surgical treatment procedure including access flap surgery, granulation tissue removal and implantoplasty combined with two different decontamination protocols and a regenerative procedure including a natural bone mineral and GTR with a collagen membrane. These authors reported clinical attachment level gains between 2.06 and 2.76 mm [19].

Mean PPD values, at the deepest site per implant, were reduced in both groups during the study period. This finding is in agreement with other studies evaluating long-term outcome of surgical peri-implantitis treatment and peri-implant defect reconstruction [20].

One implant in the PTG group, with initial, severe bone loss had a progressive radiographic bone defect after 7 years compared to baseline. The PPD registered at this site did, however, not reflect the radiographic defect depth measured, probably due to heavy calculus deposits on the implant body, which potentially may have hindered a correct probing.

Due to the removal of the prosthetic supraconstruction, access for the clinical examinations was easy at all implants. A pressure sensitive power probe was used. This resulted in minimal traumatizing of the tissue. Half of the treated implants were identified with good plaque control, which is essential for a favourable outcome.

In a study, the prognostic value of BoP [21] was evaluated related to peri-implant mucosal tissue conditions during supportive periodontal therapy. Disease progression was identified when any site bled at more than half of the recall visits over a period of 2 years. The positive predictive value for disease progression was 100% when BoP was recorded at more than 50% of recall visits. BoP was a common finding at the 7-year evaluation, and it may well be that the patients should have been followed even closer after the surgeries, and it is clearly shown that regular maintenance care is a crucial factor for lowering the risk of developing peri-implantitis [22–24].

A strict maintenance program every 3–6 months followed by a high standard of oral hygiene may hold a stable peri-implant condition after peri-implantitis surgery [16].

In the present study, access to perform sufficient plaque control at the treated implants was considered acceptable due to previous corrections of the prostheses. The frequency of supportive periodontal care (SPT) was reported to be between 6 and 12 months. Based on BoP recordings, this may not have been a sufficient regime for many of the patients in this cohort.

The patients’ general health, oral hygiene habits and compliance to the maintenance program may also affect the treatment outcome. Smoking may be a negative factor regarding complications following implant therapy [7]. In the present study, most participants were smokers or former smokers and had a history of periodontitis. No difference was noted in the number of implants with progressive bone loss versus stable conditions or between smokers and former smoker/non-smokers. This may be due to the small sample size.

In a study by Leonhardt et al. [7], nine subjects with a history of periodontitis had 26 implants diagnosed with peri-implantitis. During the 5-year follow-up after treatment, smokers with severe peri-implantitis had a less favourable treatment outcome. Another study [16] reported no difference in result between smokers and non-smokers after 2 years of follow-up.

Radiographic bone level changes for assessing the implant’s stability or progression of disease following treatment is used in several studies [7, 25]. A composite outcome regarding resolution of peri-implantitis was recommended [26].

The limitations regarding the non-standardized radiographs at the final examinations in the present study imply that results must be interpreted with care. Also, the radiopaque PTG granules were easily seen, and thereby, the cases were easily distinguished from the controls.

It has been stated that regenerative procedures do not address disease resolution but aim to fill the osseous defect [2]. Bone grafts of various materials have been used. This has classically been divided into autogenous, allogenic, xenogenic and synthetic or alloplastic materials [27].

The present study used a non-resorbable, alloplastic material (PTG) in intra-osseous defects as a reconstructed material. This technique attempts to fill the osseous defect and not solve the disease. With regard to defect fill, it is very important to keep in mind that a non-resorbable graft material such as PTG will be left unresorbed. In this study, PTG graft particles were easily seen on radiographs 7 years after therapy. Many of the particles were however scattered in the tissues around the defect. Due to the radiopaque appearance of PTG, they were easily recognized. One must in this context remember that other non-resorbable bone substitutes may also have a similar outcome but will not be as easily found and thus mistakenly considered resorbed.

Comparing the radiographs after 7 years, the extent of granules left in the osseous defects had a great variety, and two cases provided extensive loss of material. This may be explained by defect configuration, recognized at the time of surgery [28]. The inclusion of one to two walled defects may explain the absence of PTG at some sites. The PTG granules in the osseous defects may not have been well integrated with the bone, or some granules were left encapsulated in the connective tissue. Smoking habits may also have affected the reconstructive treatment [29] due to the participants who were smokers or former smokers.

None of the included subjects reported an experience of adverse effects, such as pain, discoloration of the surrounding mucosa or loose particles in conjunction with the grafting material. This is in agreement with a multicentre trial [15].

The clinical examination did not reveal any differences between the reconstructed versus non-reconstructed osseous defects related to signs of inflammation or plaque accumulations.

The present study confirmed the results from the previous study by Wohlfahrt et al. [12] and Jepsen et al. [15]. Both these publications show non-significant difference between groups in clinical parameters.

### Conclusions

This long-term follow-up of surgical treatment of peri-implant osseous defects showed unpredictable results. Loss of implants was only recorded in PTG-treated patients.
